# Comparison of robotic and open partial nephrectomy for highly complex renal tumors (RENAL nephrometry score ≥10)

**DOI:** 10.1371/journal.pone.0210413

**Published:** 2019-01-10

**Authors:** Jung Kwon Kim, Hakmin Lee, Jong Jin Oh, Sangchul Lee, Sung Kyu Hong, Sang Eun Lee, Seok-Soo Byun

**Affiliations:** Department of Urology, Seoul National University Bundang Hospital, Seongnam, Korea; University of Washington, UNITED STATES

## Abstract

**Purpose:**

To compare the outcomes of robotic partial nephrectomy (RPN) with those of open PN (OPN) in patients with highly complex renal tumors defined as RENAL nephrometry score ≥ 10

**Materials and methods:**

We analyzed clinical data from a total of 149 patients who underwent OPN or RPN for a highly complex renal mass at our institution between 2003 and 2017. Perioperative data, complication profiles, functional outcomes, pathologic variables, and oncologic outcomes were evaluated in both groups.

**Results:**

The median (interquartile range, IQR) patient age was 52.0 (42.0–59.0) years, and the median (IQR) follow-up period was 30.0 (7.0–54.0) months. Among the patients, 64 (43.0%) and 85 (57.0%) underwent OPN and RPN, respectively. The RPN group showed higher rates of clinical T1b and ≥ T2 than the OPN group (p = 0.019). There were no significant differences between the groups in terms of intraoperative outcomes such as operation time, estimated blood loss, warm ischemic time, and transfusion. Notably, the RPN group showed significantly shorter length of hospital stay than the OPN group (p < 0.001). Regarding the complication profiles and renal functional outcomes, no significant differences were reported between the groups. The estimated glomerular filtration rate decline from baseline at the last follow-up showed no significant differences between the two groups (p = 0.351). Kaplan-Meier survival analysis also showed no significant differences in survival outcomes between the groups (log-rank test, all p > 0.05).

**Conclusions:**

RPN performed in patients with highly complex renal tumors offers perioperative, functional, and oncologic outcomes comparable to those associated with OPN.

## Introduction

The current consensus guidelines recommend partial nephrectomy (PN) to be the standard treatment option for clinical T1a renal tumors [1]. A previous prospective randomized phase III study demonstrated that PN offers functional outcomes (renal function preservation) better than and cancer control comparable to radical nephrectomy (RN) in “low-stage” tumors [2]. For the last decade, with the continued development and improvement of surgical techniques, there have been trends toward using PN over RN even in larger renal tumors (≥ clinical T1b) [3–6]. However, there are still no definite consensus guidelines regarding this [7, 8]; the American Urology Association has announced that RN is the standard of care for clinical T1b renal tumors, and that PN can be performed as an alternative standard therapy in only selected patients [8].

The surgical technique of PN has evolved gradually from open PN (OPN) to laparoscopic PN (LPN), and on to robotic PN (RPN). The use of RPN has been continuously increasing with the diffusion of the da Vinci Surgical System. Patel et al. [9] reported from their population-based analysis that the use of RPN has increased from 5 to 40% between 2008 and 2011. Subsequently, RPN has broadened the spectrum of indication in large and complex tumors with its advantage of being more convenient in conducting tumor excision and renorrhaphy [10–17]. However, the majority of studies have focused dominantly on tumor size as a surrogate marker for surgical difficulty [10–14].

The RENAL nephrometry score was developed as an assessment tool for predicting surgical complexity posed by postoperative complications or warm ischemic time (WIT) [18]. This system includes five domains: Radius, Exophytic/endophytic, Nearness to collecting system or sinus, Anterior/posterior, Location relative to polar lines. Even in small renal tumors, the degree of surgical difficulty is increased in the case of high RENAL score lesions (i.e. completely endophytic, close to collecting system, posterior, entirely between polar lines). However, studies on these issues are still lacking, especially in the field of RPN [15–17]. Thus, we aimed to compare the outcomes of RPN with those of OPN in patients with highly complex renal tumors represented by RENAL nephrometry score ≥ 10.

## Materials and methods

### Ethics statement

The Institutional Review Boards of the Seoul National University Bundang Hospital approved this study (Approval number: B-1805-466-102). As the present study was carried out retrospectively, written informed consent from patients was waived. Personal identifiers were completely removed and the data were analyzed anonymously. Our study was conducted according to the ethical standards recommended by the 1964 Declaration of Helsinki and its later amendments.

### Study cohort

From June 2003 to March 2017, a total of 161 patients who underwent OPN or RPN for a highly complex renal mass (RENAL score ≥ 10) at our institution were included in this retrospective study. RPNs were performed after 2008 using the da Vinci Surgical System. Lymph node dissection was performed in the case of suspicious findings indicating lymph node invasion in preoperative imaging and/or intraoperative findings. Patients were excluded if they had non- renal cell carcinoma malignancies or metastatic disease. We also excluded the patients with a solitary kidney, multifocal tumors, von Hippel-Lindau syndrome, or OPN under hypothermia and cold ischemia. Subsequently, 7 patients of RPN group and 5 patients of OPN group were excluded; a total of 149 patients were included in final analysis.

### Acquisition and definition of data

Clinical data in the prospectively maintained database of our institution were retrospectively reviewed. The RENAL nephrometry score was calculated by each physician as previously described [18]. The clinical variables measured as baseline characteristics according to the type of surgeries (OPN vs. RPN) included age, sex, body mass index (BMI), past medical history (including diabetes mellitus [DM], hypertension [HTN], and chronic kidney disease [CKD]), Eastern Cooperative Oncology Group performance status, American Society of Anesthesiologists scores, Charlson comorbidity index, laboratory data (including serum creatinine and an estimated glomerular filtration rate [eGFR] calculated by Modification of Diet in Renal Disease equation [19]), tumor laterality, clinical stage, and RENAL nephrometry score. Tumor size was determined as the longest diameter of each tumor in any single plane of the preoperative imaging study.

Variables for perioperative outcome analysis included operation time, estimated blood loss (EBL), WIT, length of hospital stay (LOS), intra-/postoperative transfusion, complication profiles including Clavien grade [20], and renal function changes. De novo CKD was defined as the development of stage ≥ 3 CKD with two consecutive values of eGFR < 60 ml/min/1.73m^2^ [21].

Pathological parameters including histological type according to the World Health Organization (WHO) classification system [22], pathologic stage according to the 7th edition of American Joint Committee guidelines [23], Fuhrmann nuclear grade, and positive surgical margin (PSM) were also evaluated.

Recurrence was defined as radiographically verified distant metastasis or local disease recurrence during the study period.

### Follow-up protocol

According to the institutional standardized postoperative protocol, patients were generally followed-up after surgery at least every six months in the first year, annually over the next four years, and every two years thereafter. Follow-up protocols consisted of computed tomography (CT) or magnetic resonance imaging (MRI), bone scan, and chest radiography (and/or chest CT).

Recurrence-free survival (RFS) was defined as the interval between the date of surgery and the time of first tumor recurrence. The cause of death was determined by the responsible physicians and death certificates. Overall survival (OS) was calculated from the date of surgery to the date of last follow-up or death.

### Statistical analyses

Clinicopathological characteristics were compared between the OPN and RPN groups using a chi-squared test for categorical variables, and an independent t-test or Mann-Whitney U test for continuous variables. Kaplan-Meier curve analysis was used to calculate the survival estimates for RFS and OS, and the log-rank test was used to conduct comparisons between the groups. All statistical analyses were performed using commercially available software (IBM SPSS Statistics ver. 21.0, Armonk, NY, USA) and two-sided p values < 0.05 were considered statistically significant.

## Results

The median (IQR) patient age was 52.0 (42.0–59.0) years, and the median (IQR) follow-up period was 30.0 (7.0–54.0) months. At the last follow-up, there were three (2.0%) patients who had died of any cause (one, other cancer-related death [ampulla of vater cancer]; two, death with unknown cause from death certificates), and recurrence occurred in six (3.8%) patients overall. Among all patients, 64 (43.0%) underwent OPN and 85 (57.0%) underwent RPN.

### Comparison of baseline characteristics

Comparative analysis results of the preoperative clinical features between the two groups are summarized in Table 1. The RPN group had a higher rate of clinical stage T1b and ≥T2 compared with the OPN group (p = 0.019). However, there were no significant differences in the other variables, notably in terms of preoperative renal function profile (serum creatinine and eGFR) and RENAL nephrometry score.

**Table 1:** Baseline characteristics.

Variables	Median (interquartile range) or counts (%)			P
	Total (N = 149)	OPN (N = 64)	RPN (N = 85)	
Age, years	52.0 (42.0–60.0)	52.0 (40.5–60.5)	53.0 (42.0–60.0)	0.947
Sex, male	97 (65.1%)	42 (65.6%)	55 (64.7%)	1.000
BMI, kg/m^2^	24.7 (22.9–27.1)	24.8 (22.9–26.5)	24.7 (22.9–27.5)	0.969
ECOG score, ≥1	3 (2.0%)	2 (3.1%)	1 (1.2%)	0.577
ASA score, ≥2	4 (2.7%)	2 (3.1%)	2 (2.4%)	0.897
CCI score	2 (1–3)	2 (1–3)	2 (1–2)	0.642
Diabetes mellitus, yes	19 (12.8%)	5 (7.8%)	14 (16.5%)	0.141
Hypertension, yes	53 (35.6%)	20 (31.3%)	33 (38.8%)	0.389
*CKD, stage≥3	6 (4.0%)	3 (4.7%)	3 (3.5%)	1.000
Preoperative creatinine, mg/dL	0.88 (0.72–1.00)	0.91 (0.77–1.00)	0.86 (0.69–0.96)	0.842
Preoperative eGFR, ml/min/1.73 m^2^	85.6 (76.4–101.5)	83.3 (74.2–98.5)	87.1 (79.8–103.8)	0.085
Clinical stage				0.019
T1a	78 (52.3%)	42 (65.6%)	36 (42.4%)	
T1b	51 (34.2%)	16 (25.0%)	35 (41.2%)	
≥ T2	20 (13.4%)	6 (9.4%)	14 (16.5%)	
RENAL score	10.0 (10.0–10.0)	10.1 (10.0–10.0)	10.2 (10.0–10.0)	0.346
Laterality, Lt.	85 (57.0%)	36 (56.3%)	49 (57.6%)	0.869

ASA, American Society of Anesthesiologists; BMI, body mass index; CCI, Charlson comorbidity index; CKD, chronic kidney diseas; ECOG, Eastern Cooperative Oncology Group; eGFR, estimated glomerular filtration rate; OPN, open partial nephrectomy; RPN, robotic partial nephrectomy

*GFR < 60 ml/min/1.73m^2^

### Comparison of perioperative outcomes

There were no significant differences in terms of intraoperative outcomes including operation time, EBL, WIT, and transfusion (Table 2). Notably, the RPN group showed significantly shorter LOS than the OPN group (median, 5 [RPN] vs. 7 [OPN] days, p < 0.001, Table 2). In complication profiles, the RPN group showed the lower major (Clavien grade 3–5) complication rates in the early (within three months of surgery) postoperative periods, but this trend was not statistically significant (9.4% [RPN] vs. 14.1% [OPN], p = 0.440, Tables 2 and 3). Regarding functional outcomes, the mean value of eGFR decline from baseline at the last follow-up showed no significant differences between the two groups (mean, 6.5 [RPN] vs. 3.8 [OPN] ml/min/1.73 m^2^, p = 0.351, Table 4). In addition, there was no significant difference in the development of de novo CKD (p = 1.000).

**Table 2:** Perioperative outcomes.

Variables	Median (interquartile range) or counts (%)		P
	OPN (N = 64)	RPN (N = 85)	
Operation time, min	145 (105–180)	150 (110–190)	0.709
Estimated blood loss, ml	200 (100–300)	200 (100–300)	0.888
Warm ischemic time, min	21 (18–30)	24 (19–34)	0.147
Transfusion			
Intraoperative	3 (4.8%)	3 (3.5%)	0.700
Postoperative	4 (6.3%)	5 (5.9%)	1.000
Intraoperative complications	4 (6.3%)	8 (9.4%)	0.556
Postoperative complications			
Overall (Clavien 1–5), n (%)	15 (23.4%)	16 (18.8%)	0.544
Major (Clavien 3–5), n (%)	9 (14.1%)	8 (9.4%)	0.440
Length of hospital stay, day	7 (5–9)	5 (5–7)	<0.001
VAS score for pain in postoperative 1 day	4 (4–5)	4.5 (4–5)	0.439

OPN, open partial nephrectomy; RPN, robotic partial nephrectomy; VAS, visual analogue scale

**Table 3:** Complication profiles.

Variables	OPN (N = 64)	RPN (N = 85)	P
Intraoperative complications	4 (6.3%)	8 (9.4%)	0.556
Postoperative complications			
Overall (Clavien 1–5), n (%)	18 (28.1%)	18 (21.2%)	0.548
Major (Clavien 3–5), n (%)	12 (18.8%)	10 (11.8%)	0.438
Details			
Wound dehiscence	3	2	
Postoperative bleeding	9	4	
Pseudoaneurysm	3	2	
Pneumonia	1	-	
Atelectasis / Desaturation	1	-	
Pneumothorax	1	2	
Acute renal failure	-	2	
Ileus	-	2	
Urinary retention	-	2	
Other infection	-	2	

**Table 4:** Renal functional outcomes.

Variables	OPN (N = 64)	RPN (N = 85)	P
Preoperative creatinine, mg/dL	0.91 (0.77–1.00)	0.86 (0.69–0.96)	0.842
Preoperative eGFR, ml/min/1.73 m^2^	83.3 (74.2–98.5)	87.1 (79.8–103.8)	0.085
*CKD, stage≥3	3 (4.7%)	3 (3.5%)	1.000
Latest postoperative creatinine, mg/dL, median (IQR)	0.92 (0.74–1.06)	0.87 (0.71–1.05)	0.662
Latest postoperative eGFR, ml/min/1.73 m^2^, median (IQR)	82.4 (69.9–92.3)	84.8 (69.1–84.8)	0.335
^+^eGFR decline from baseline, mean (SD)	3.8 (16.6)	6.5 (18.0)	0.351
De novo CKD, stage≥3	2 (3.3)	4 (4.9)	1.000
Follow-up duration, months, median (IQR)	53.0 (33.3–81.0)	15.0 (5.5–33.0)	<0.001

CKD, chronic kidney diseas; eGFR, estimated glomerular filtration rate; OPN, open partial nephrectomy; RPN, robotic partial nephrectomy

*GFR < 60 ml/min/1.73m^2^, ^+^ paired T-test

### Comparison of pathologic and oncologic outcomes

**Fig 1:**
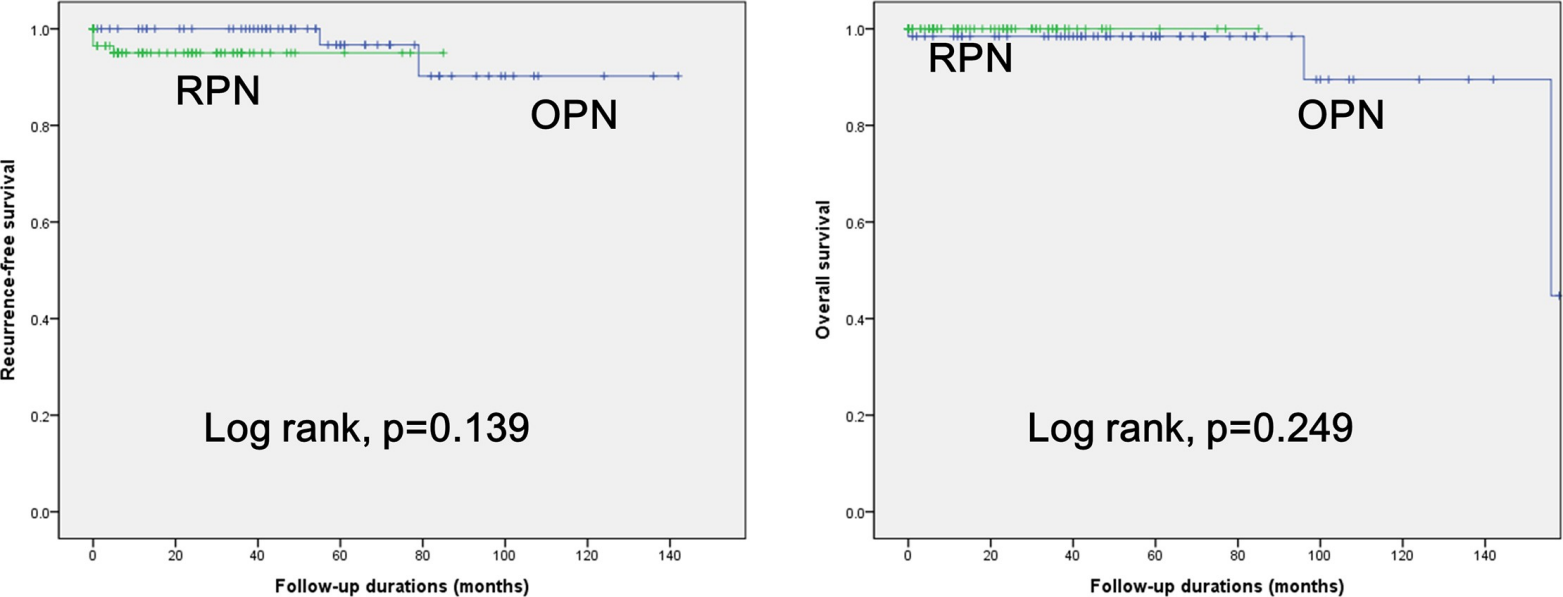
Kaplan-Meier curve analysis of (A) recurrence-free survival and (B) overall survival according to the type of surgery performed.

The RPN group showed a significantly larger pathologic tumor size than the OPN group (median, 4.3 vs. 3.1 cm, p = 0.014). There were no significant differences in the other pathologic outcomes including pathologic stage, Fuhrmann nuclear grade, and histologic subtypes (Table 5). There was no PSM in the RPN group. In addition, Kaplan-Meier survival analysis showed no significant differences in RFS and OS between the two groups (log-rank test, all p > 0.05, Fig 1).

**Table 5:** Pathologic and oncologic outcomes.

Variables	Median (interquartile range) or counts (%)		P
	OPN (N = 64)	RPN (N = 85)	
Tumor size (cm)	3.1 (2.3–4.8)	4.3 (2.9–5.2)	0.014
Pathological stage	4 (6.3%)	1 (1.2%)	0.165
pT1	57 (89.1%)	74 (87.1%)	
pT2	3 (4.7%)	10 (11.8%)	
pT3	4 (6.3%)	1 (1.2%)	
Fuhrmann grade			0.386
≤ 2	36 (56.3%)	40 (47.1%)	
≥ 3	28 (43.8%)	45 (52.9%)	
Histological subtype			0.203
Clear cell	50 (78.1%)	65 (76.5%)	
Papillary	3 (4.7%)	1 (1.2%)	
Chromophobe	3 (4.7%)	10 (11.8%)	
Collecting duct	0 (0%)	0 (0%)	
Unclassified	0 (0%)	2 (2.4%)	
Benign	8 (12.5%)	7 (8.2%)	
Positive surgical margin	1 (1.6%)	0 (0%)	0.432
Recurrence	2 (3.1%)	4 (4.7%)	0.306
Overall mortality	3 (4.7%)	0 (0%)	0.077
Follow-up duration, months	53.0 (33.3–81.0)	15.0 (5.5–33.0)	<0.001

OPN, open partial nephrectomy; RPN, robotic partial nephrectomy

## Discussion

The RENAL nephrometry scoring system categorizes the complexity of renal masses. The score range of 4–6, 7–9, and ≥ 10 are deemed as low, moderate, and high complexity lesions, respectively [18]. Regarding complex renal tumors defined as RENAL nephrometry score ≥ 7, several previous studies reported that RPN offers perioperative, functional and oncological outcomes comparable to those associated with LPN or OPN [15–17]. Long et al. [16] compared perioperative outcomes between the LPN and RPN group for a complex renal tumors of RENAL nephrometry score ≥ 7 in a large single center cohort. Consequently, they found that LPN was associated with a higher conversion rate to RN (11.5% vs. 1%, p < 0.001) and a higher decrease in eGFR (-16.0% vs. -12.6%, p = 0.03). However, there were no significant differences in perioperative outcomes posed by WIT, EBL, transfusion rate, or complications between the two groups. In a very recent meta-analysis, Cacciamani et al. [24] reported that all perioperative, oncological, and survival outcomes were similar between OPN and RPN groups with similar RENAL nephrometry score. Importantly, in their sensitivity analyses focusing only on complex renal masses defined as RENAL nephrometry score ≥ 7, RPN had lesser EBL (Weighted mean difference [WMD], 66.32; 95% confidence interval [CI], 26.06–106.58; p = 0.001), fewer overall postoperative complications (Odd ratio [OR], 2.15; 95% CI, 1.40–3.29; p = 0.0004) and shorter LOS (WMD, 1.57, 95% CI, 1.04–2.09; p < 0.00001).

In a recent study from Cleveland Clinic, Garisto et al. [25] represented the first series comparing OPN (N = 76) vs. RPN (N = 203) for patients with RENAL nephrometry score ≥ 10, which is deemed as highly complex lesion [18]. The authors demonstrated that RPN was associated with a lower EBL (200 vs. 300ml, p < 0.001), shorter ischemic time (28 vs. 37 min, p < 0.001), lower intraoperative transfusion rates (3% vs. 15.8%, p < 0.001), and shorter LOS (3 vs. 5 days, p < 0.001) compared to OPN. Regarding renal functional outcomes, the median (IQR) value of eGFR at 3^rd^, 6^th^, and 12^th^ postoperative months showed no significant differences between the two groups. Accordingly, CKD upstaging rates were comparable (44.3% [RPN] vs. 47.4% [OPN], p = 0.643). They also found no significant differences between the groups in survival outcomes including OS and RFS.

In the current study, we also found that the RPN group showed significantly shorter LOS (median, 5 vs. 7 days, p < 0.001, Table 2) in comparison with the OPN group. However, we found no significant differences between the groups in terms of intraoperative outcomes such as operation time, EBL, WIT, and transfusion rates (Table 1). Also, the mean value of eGFR decline at the last follow-up and the development of de novo CKD showed no significant differences between the two groups (Table 4).

Up to now, over 1000 cases of RPN have been performed in our institution, and recently, vast majority of renal tumor cases were performed by RPN (S1 Fig). In 2017, we conducted 90 cases of RN and 241 cases of PN; and PN comprised 222 cases of RPN, 9 cases of LPN, and 10 cases of OPN. With this broadening indication for RPN even in larger tumors, higher clinical stage tumors were dominantly included in the RPN groups. Consequently, current study showed a higher rate of clinical stage T1b and ≥ T2 in the RPN group compared to the OPN group (p = 0.019, Table 1). With this perspective, the median (IQR) WIT of the RPN group was slightly longer than that of the OPN group (median, 24 vs. 21 min, p = 0.147); this is significantly longer than the 17–20 min reported in previous RPN series [26–30]. In a previous study on Korean population, Kang et al. [27] reported that T1b cases showed longer operative times and WIT than T1a cases in 362 patients underwent RPN. Generally, in patients with highly complex tumors, such as those that make up our cohort, the longer WIT would be inevitable [12, 14, 31]; instead, it would be better to focus on the other outcomes such as renal functional outcomes and complication profiles. Importantly, there were no significant differences between the groups in terms of mean eGFR decline, De novo CKD, and intra/postoperative complication rates (Tables 2–4).

Regarding the pathologic and oncologic outcomes, we found no significant differences between the groups (Table 5 and Fig 1). There was no PSM in the RPN group. In fact, oncologic outcomes are of the most concern in the surgical approach to renal tumors. Several previous studies reported survival outcomes in the RPN group comparable or even superior to those in LPN or OPN groups in patients with complex renal tumors [11–17]. Cacciamani et al. [24] reported in their recent meta-analysis study that RPN was superior for PSM (OR, 1.73; p < 0.0001) and OS (OR, 2.98; p = 0.04). However, in the majority of studies, the follow-up duration was too short to draw definitive conclusions. Even in a recent study from Cleveland Clinic, the median follow-up period was only 25 months [25]. The current study also demonstrated that the RPN group had a significantly shorter follow-up duration than the OPN group (median, 15.0 vs. 53.0 months, p<0.001). Therefore, we could not draw definitive conclusions based on the present study. Long-term follow-up studies with larger sample sizes are needed to verify these results.

With the wide spread of RPN in field of renal tumors, the cost-effectiveness of RPN has been a source of unresolved debate [32, 33]. In a recent study, Buse et al. [32] described the results of cost-effectiveness analysis of RPN vs. OPN in US. The mean in‐hospital costs were $14,824 (95% CI, $13,368-$16,898) for RPN and $15,094 (95% CI, $13,491-$17,140) for OPN. Complications after RPN occurred in 23.3% (95% CI, 20.0–25.8%) and after OPN in 36.1% (95% CI, 35.6–36.6%) of the patients. In a sensitivity analysis, limited center experience was associated with relevant increase in RPN cost and consequently in low cost‐effectiveness. Accordingly, they concluded that RPN resulted in nominally lower cost but fewer perioperative complications than OPN, and RPN was not cost-effective in less experienced centers. In 2015, we also analyzed the difference between costs and utility after one year of RPN through incremental cost-effectiveness ratio (ICER) using a Decision Tree model [not published data]. Consequently, we found that ICER was 130 million KRW (willingness to pay [WTP]: 30 million KRW per 1 Quality-adjusted life year [QALY]); it is not cost-effective compared to laparotomy. However, as previously described, we found that the RPN group showed comparable or superior perioperative outcomes compared to the OPN group (Tables 2–4). From there results, we tentatively concluded that RPN should be considered especially in patients with highly complex renal tumors in high-volume centers.

The current study has several limitations. First, even with a large tertiary center cohort, the study population was still small due to the rarity of highly complex renal tumors (RENAL nephrometry score ≥ 10). Accordingly, the events of recurrence and mortality were rare, which prevented a clear analysis of RFS and OS. In addition, each case of RPN was performed after 2008 (toward the latter half of the study period); accordingly, the results may have been affected by each surgeon’s learning curve and RPN experience. In fact, the majority of cases were performed by two surgeons (S.E.L. and S.S.B.) with extensive and high-volume robotic experience; some of the cases (9.4%) were performed by a single low-volume surgeon. This surgeon-related factor may certainly have biased some part of the perioperative outcomes. Notably, in subsequent subgroup analysis of perioperative outcomes between high and low volume surgeons, there were significant differences between the groups in terms of operation time, EBL, WIT, and intraoperative transfusion rate (S1 Table). However, we found no significant changes even after excluding the data of a low volume surgeon in perioperative outcomes (S2 Table). Regarding this issue, it is important to note that these study findings should not be generalized to the entire urological community; RPN is reserved for experienced robotic surgeons and high-volume centers of excellence.

## Conclusion

RPN performed in patients with highly complex renal tumors (RENAL nephrometry score ≥ 10) offers perioperative, functional, and oncologic outcomes comparable to those associated with OPN. Subsequently, we can extrapolate to suggest that the indication for RPN is broadening for all renal tumors, regardless of surgical difficulty. Longer follow-up studies with larger ample sizes and randomized controlled trials are awaited to verify these results.

## Supporting information

S1 Dataset De-identified data set. (XLSX)

S1 Fig Number of partial nephrectomies in Seoul National University Bundang Hospital according to the surgical modalities. (TIF)

S1 Table Comparative analysis of perioperative outcomes between high and low volume surgeons. (PDF)

S2 Table Perioperative outcomes after excluding the data of low volume surgeon. (PDF)
