# Supplementary figures and images for: Comparison of robotic and open partial nephrectomy for highly complex renal tumors (RENAL nephrometry score ≥10)
Source: PLoS One. 2019 Jan 10;14(1):e0210413. doi: 10.1371/journal.pone.0210413 (PMC6328203; doi:10.1371/journal.pone.0210413)

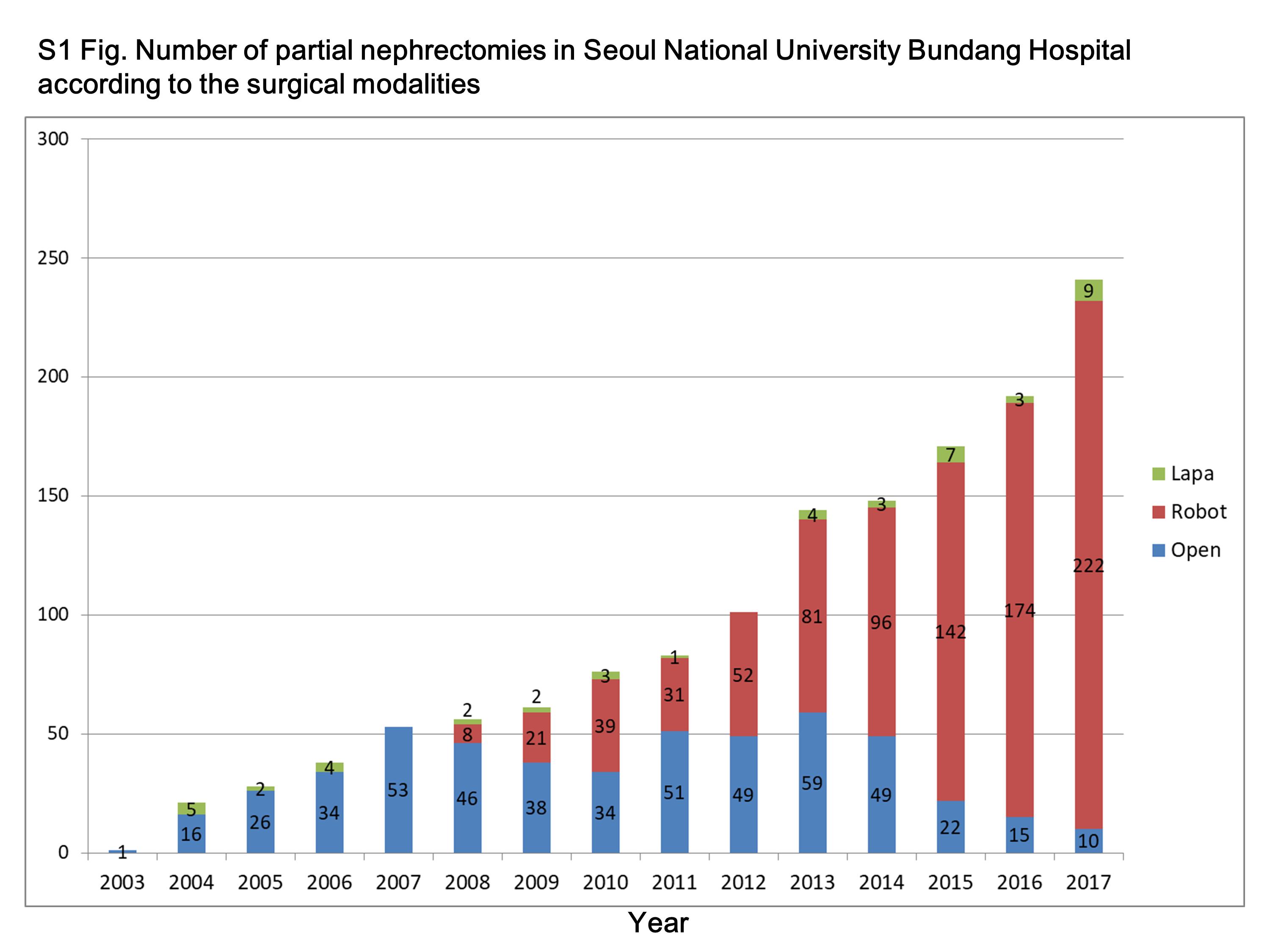

Supplement: S1 Fig — (TIF) [file pone.0210413.s002.tif]
