# Supplementary material for: Comparison of robotic and open partial nephrectomy for highly complex renal tumors (RENAL nephrometry score ≥10)
Source: PLoS One. 2019 Jan 10;14(1):e0210413. doi: 10.1371/journal.pone.0210413 (PMC6328203; doi:10.1371/journal.pone.0210413)
Supplement: S1 Table — (PDF) [file pone.0210413.s003.pdf]

S1 Table. Comparative analysis of perioperative outcomes between high and low volume surgeons

Variables	Mean (SD) or counts (%)		P
	High volume surgeons (N=77)	Low volume surgeon (N=8)	
Operation time, min	149.1 (58.4)	210.7 (78.7)	0.011
Estimated blood loss, ml	206.6 (174.1)	571.4 (461.8)	<0.001
Warm ischemic time, min	26.4 (10.4)	38.8 (14.7)	0.008
Transfusion			
Intraoperative	1 (1.3%)	2 (25.0%)	0.022
Postoperative	4 (5.2%)	1 (12.5%)	0.398
Intraoperative complications	6 (7.8%)	2 (25.0%)	0.163
Postoperative complications			
Overall (Clavien 1-5), n (%)	14 (18.2%)	2 (25.0%)	0.639
Major (Clavien 3-5), n (%)	8 (10.4%)	0 (0)	0.338
Length of hospital stay, day	5.1 (1.2)	5.1 (0.9)	0.781
VAS score for pain in postoperative 1 day	4.4 (0.9)	4.5 (1.1)	0.698
eGFR decline from baseline, ml/min/1.73 m^2	6.9 (17.6)	2.4 (22.3)	0.503
