# Supplementary material for: Comparison of robotic and open partial nephrectomy for highly complex renal tumors (RENAL nephrometry score ≥10)
Source: PLoS One. 2019 Jan 10;14(1):e0210413. doi: 10.1371/journal.pone.0210413 (PMC6328203; doi:10.1371/journal.pone.0210413)
Supplement: S2 Table — (PDF) [file pone.0210413.s004.pdf]

S2 Table. Perioperative outcomes after excluding the data of low volume surgeon

Variables	Mean (SD) or counts (%)		P
	OPN (N=64)	RPN, high volume (N=77)	
Operation time, min	150.5 (61.5)	149.2 (58.4)	0.895
Estimated blood loss, ml	234.7 (177.2)	206.6 (174.1)	0.356
Warm ischemic time, min	24.6 (10.7)	26.4 (10.4)	0.335
Transfusion			
Intraoperative	3 (4.8%)	1 (1.3%)	0.221
Postoperative	4 (6.3%)	4 (5.2%)	0.770
Intraoperative complications	4 (6.3%)	6 (7.8%)	0.722
Postoperative complications			
Overall (Clavien 1-5), n (%)	15 (23.4%)	14 (18.2%)	0.442
Major (Clavien 3-5), n (%)	9 (14.1%)	8 (10.4%)	0.505
Length of hospital stay, day	6.8 (2.1)	5.1 (0.8)	<0.001
VAS score for pain in postoperative 1 day	4.2 (1.3)	4.4 (0.9)	0.493
eGFR decline from baseline, ml/min/1.73 m^2	3.8 (16.6)	6.8 (17.6)	0.284
